# Measurement of Creep Stress Exponent of TC17 Titanium Alloy by Nanoindentation Method at Room Temperature

**DOI:** 10.3390/ma16165702

**Published:** 2023-08-20

**Authors:** Fagui Li, Xiyu Chen, Yuannan Wang, Guolong Zhao, Yinfei Yang

**Affiliations:** 1 College of Mechanical and Electrical Engineering, Nanjing University of Aeronautics & Astronautics, Nanjing 210016, China; lifagui@nuaa.edu.cn (F.L.); wangyuannan@mindray.com (Y.W.); zhaogl@nuaa.edu.cn (G.Z.); 2 Nanjing High Speed & Accurate Gear (Group) Co., Ltd., Nanjing 211100, China; ycx123146179@163.com

**Keywords:** TC17, creep, nanoindentation, creep stress exponent

## Abstract

The creep stress exponent is commonly employed to characterize the deformation mechanism during the steady-state creep stage, serving as an indicator of creep behavior. The creep phenomenon of high melting point metallic materials is not obvious at room temperature. However, the nanoindentation method proves suitable for investigating the creep properties of metallic materials under such conditions. Consequently, this paper places emphasis on measuring the creep stress exponent of TC17 titanium alloy at room temperature using the load preservation stage of the nanoindentation method with a constant loading rate. In order to investigate the effects of loading rate and maximum load on the experimental results, different loading rates were applied to the diamond Berkovich indenter to reach different maximum loads. The indenter was held under the maximum load for a duration of 360 s, and the relationship between the indentation strain rate and indentation stress during the holding process was used to obtain the creep stress exponent of the material at room temperature. The findings indicate that within the loading rate range of 1.25 to 15 mN/s and maximum load range of 50 to 300 mN, the influence on the experimental results is insignificant. Ultimately, the distribution range of the creep stress exponent for TC17 titanium alloy at room temperature was measured to be 8.524–8.687.

## 1. Introduction

Titanium alloys have garnered significant attention due to their remarkable properties, including high specific strength, low density, excellent corrosion resistance, impressive thermal stability, and high fracture toughness. Consequently, these alloys have found extensive applications in diverse sectors such as nuclear plants, aerospace, the chemical industry, and biomedicine [1,2,3]. The nominal composition of TC17 titanium alloy is mainly Ti-5Al-4Mo-4Cr-2Sn-2Zr, and it is a kind of α+β type two-phase titanium alloy material, rich in β stable phase. TC17 titanium alloy has the advantages of high strength, high toughness, high fracture toughness, high hardenability and a wide forging temperature range. Hence, it is suitable for the manufacture of aircraft engine compressor blades/disks [4]. At present, research on TC17 titanium alloy, both domestically and internationally, has mainly concentrated on its microstructure, mechanical properties [5,6,7], and thermal deformation behavior [8]. However, it is equally crucial to investigate the creep properties of the TC17 titanium alloy from an engineering application standpoint.

Creep refers to the gradual plastic deformation of metal over an extended period under constant temperature and load [9,10]. This phenomenon arises due to the sustained application of stress below the material’s yield strength. Under the continuous action of the operating temperature and external stress, the material will produce permanent deformation, which cannot be restored with the extension of time. This phenomenon is known as creep deformation [11,12]. The creep deformation can be influenced by numerous factors during the creep process. These factors include macroscopic conditions, such as the internal organization of the material and grain boundaries, etc. [13,14,15,16], as well as microscopic conditions, including thermal vibration and atomic diffusion [17].

The creep behavior of materials can be divided into three stages: transient creep, steady-state creep and accelerated creep stage [18]. The transient creep stage refers to the initial stage of creep, where the material exhibits a high creep rate, which gradually decreases over time. This phenomenon is attributed to work hardening. The creep rate of the material is basically unchanged in the steady-state creep stage, which is due to the hardening and softening to maintain a balance. In the accelerated creep stage, the creep rate increases rapidly over time, and fracture occurs after reaching the critical value, which is due to the continuous accumulation of defects in the microstructure. There are generally three commonly used tensile creep mechanisms for materials [19]: (1) Dislocation movement mechanism: Dislocation slip is an important cause of plastic deformation in materials. At room temperature, dislocation slip is resisted and stagnant, making the creep deformation rate slow down. However, under high-temperature conditions, it continues by climbing, thus accelerating creep deformation. (2) Diffusion creep mechanism: When subjected to external forces, grain boundaries experience the formation of vacancies under tensile stress. Conversely, when exposed to compressive stress, the concentration of vacancies at the grain boundaries is minimal. This discrepancy in vacancy concentration prompts the migration of vacancies towards the grain boundaries experiencing compressive stress, consequently causing creep elongation. (3) Grain boundary sliding mechanism: It must be coordinated with the diffusion creep mechanism for the material to deform continuously. At the intersection of three grain boundaries, grain boundary movement can lead to larger stress concentrations, thus inducing cracks. In some occasions with high precision requirements, the creep phenomenon of materials must be considered. Moreover, for metal materials with high melting points, the creep phenomenon is not obvious, and the creep deformation is slow at room temperature. Compared with high temperatures, it takes more time to measure the creep behavior at room temperature.

In recent years, the nanoindentation method has been widely employed to investigate the mechanical properties of materials. This technique allows for the determination of various parameters, including the load–displacement curve, hardness, fracture toughness, and creep [20]. Compared to the tensile test method, the nanoindentation technique offers several advantages. It requires simpler sample preparation, features shorter test cycles, provides higher accuracy and repeatability when applied to the sample, and allows for the investigation of creep behavior in localized regions at room temperature. The nanoindentation method can apply a constant load to the indenter without damaging the material. This allows for the collection of load, displacement, and time data during both the loading and unloading phases, ultimately enabling the determination of the material’s creep curves.

In the traditional tensile test, the creep strain rate in the steady-state creep stage of materials can be described by the Dorn power law relationship [21]. Whether the relevant parameters of the constitutive equation obtained using nanoindentation technology are consistent with the traditional tensile creep test is of great significance for studying the correlation between them. Therefore, many researchers have made a lot of attempts. R. Mahmudi compared the creep stress exponent of Pb-Sn alloy as consistent [22]. In the nanoindentation experiment of conical and triangular pyramid indenters, the stress and strain of the material have certain self-similarity. Although the stress and strain at different points will change during the deformation process, the ratio will remain the same. Based on this assumption, Sargent and Ashby [23] finally concluded that for indentation of different sizes, the specific equivalent stress and equivalent strain rate of the material follow the Dorn power law relationship.

Currently, conventional uniaxial tensile and nanoindentation methods are predominantly employed to investigate the creep properties of metallic materials. The conventional uniaxial tensile method is primarily applicable for studying the high-temperature creep behavior of metallic materials, while the creep phenomenon in metallic materials with high melting points is less discernible at room temperature. Furthermore, when using the uniaxial tensile method, it is difficult to determine the creep parameters of materials at room temperature and necessitates significant time and resources. In contrast, the nanoindentation method is suitable for investigating the creep properties of metallic materials under room temperature conditions. Therefore, nanoindentation serves as a more effective approach to exploring the room-temperature creep properties of metallic materials with high melting points. Additionally, there is limited research available on the room temperature creep properties of TC17 titanium alloy. Hence, this paper aims to utilize the nanoindentation method to examine the creep properties of TC17 titanium alloy at room temperature.

## 2. Experimental Procedures

### 2.1. Test Sample and Equipment

The material used in the test is TC17 titanium alloy, which was processed into rectangular pieces with dimensions of 15 mm × 15 mm × 5 mm through wire cutting. The chemical compositions (wt.%) of the alloy are shown in Table 1. The sample was first mechanically polished step by step with sandpaper of gradually finer grain size and then polished with diamond polishing paste. Finally, it was cleaned via ultrasonication. The surface roughness of the sample was measured using an atomic force microscope as being 63–69 nm, ensuring the surface roughness and flatness of the sample.

**Figure 1:**
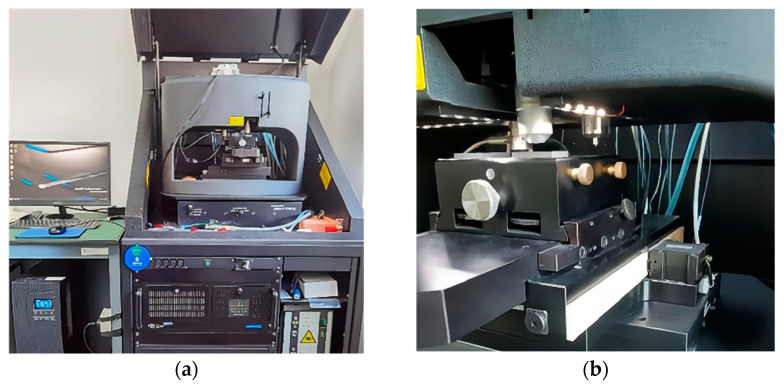
The nanoindentation test equipment: (**a**) Nanoindentation equipment and control system; (**b**) Nanoindentation process.

The indentation test equipment is a NANOG 200 nanoindentation tester produced by MTS Company in the U.S.A, as shown in Figure 1. Nano Indenter G200 was used in the experiment, and it was a diamond Berkovich type. The resolution of displacement and load of the equipment were 0.01 nm and 50 nN, respectively.

### 2.2. Nanoindentation Test Procedures

**Figure 2:**
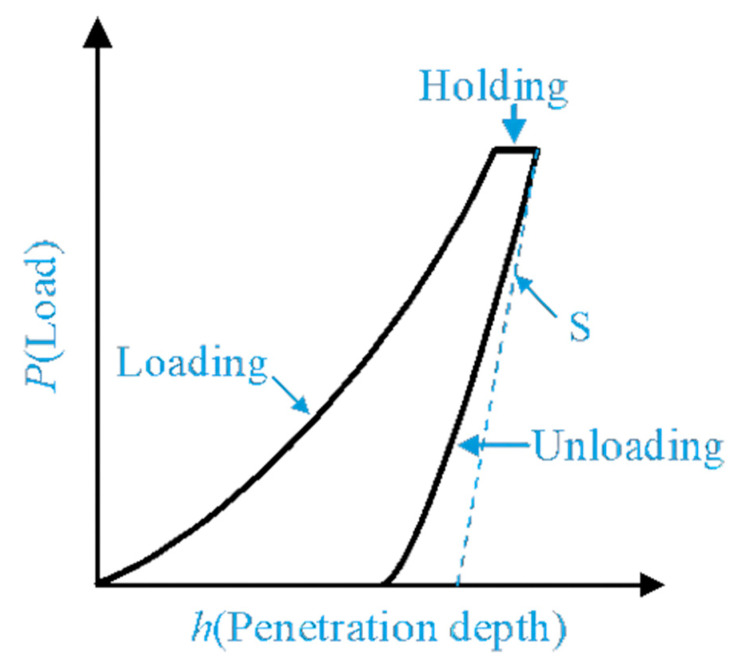
The typical loading-holding-unloading curves.

The indentation process of the indentation creep test is mainly divided into three stages: loading, holding and unloading. A typical loading–holding–unloading curve is shown in Figure 2. The creep properties of materials can be measured in the holding stage. The variation of displacement during the holding stage is the amount of creep displacement change in the material, and the holding time represents the creep time.

To account for the size effect of the indentation, the maximum load for the indentation test of TC17 alloy before the formal start of the creep test was selected at various values: 0.01 mN, 0.1 mN, 0.5 mN, 5 mN, 10 mN, 25 mN, 50 mN, 150 mN, 300 mN, and 500 mN.

**Figure 3:**
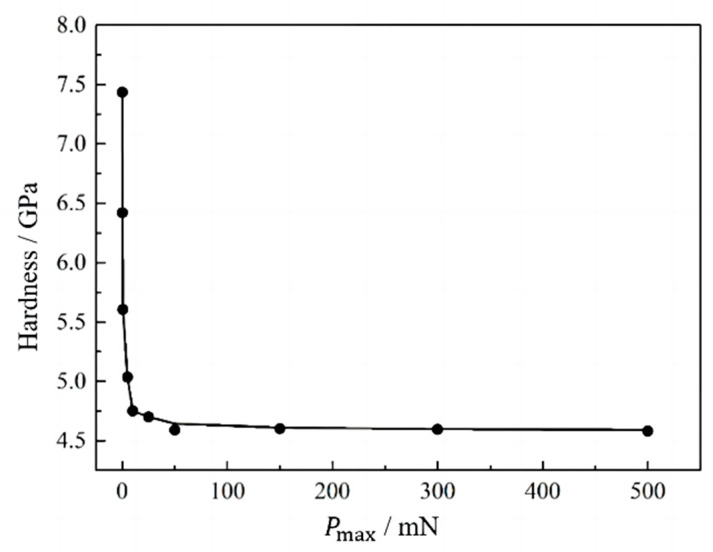
Indentation load-hardness curve.

Figure 3 shows the relationship between the variation in indentation hardness and the maximum load. The indentation hardness decreases with the increase in maximum indentation load. When the maximum indentation load reaches about 50 mN, the indentation hardness starts to stabilize. Therefore, the maximum indentation load is selected to be more than 50 mN.

#### Nanoindentation Test Procedures at Room Temperature

The process of nanoindentation creep test at room temperature is as follows:

(1) During the test, the indenter first approached the sample surface slowly at a rate of 10 nm/s. When the contact stiffness suddenly increased, it indicated that the indenter contacted the surface.

(2) Loading stage: The material was loaded to the maximum load $P_{max}$ using the constant loading rate method. In order to obtain the creep stress exponent of TC17 at room temperature, the maximum loads selected were 50 mN, 100 mN, 200 mN, and 300 mN, respectively, and the loading time was set at 20 s and 40 s, respectively.

(3) Holding stage: The maximum load was kept for 360 s to obtain the creep characteristics of the material.

(4) Unloading stage: After unloading at the same loading rate, when unloading to 0.1 $P_{max}$, maintained pressure for a period of time to eliminate the influence of thermal drift. Finally, the indenter was withdrawn from the specimens’ surface to terminate the creep test.

The whole experiment was carried out at room temperature with temperature fluctuation less than ±2 °C. Each condition was repeated three times, and the average value was taken as the final experiment result to improve the accuracy of the experiment. The distance between all adjacent indentation positions in the above tests exceeded 200 μm so as to avoid material stress near the indentation affecting the results of the new indentation, which would result in the inaccuracy of the test results.

**Figure 4:**
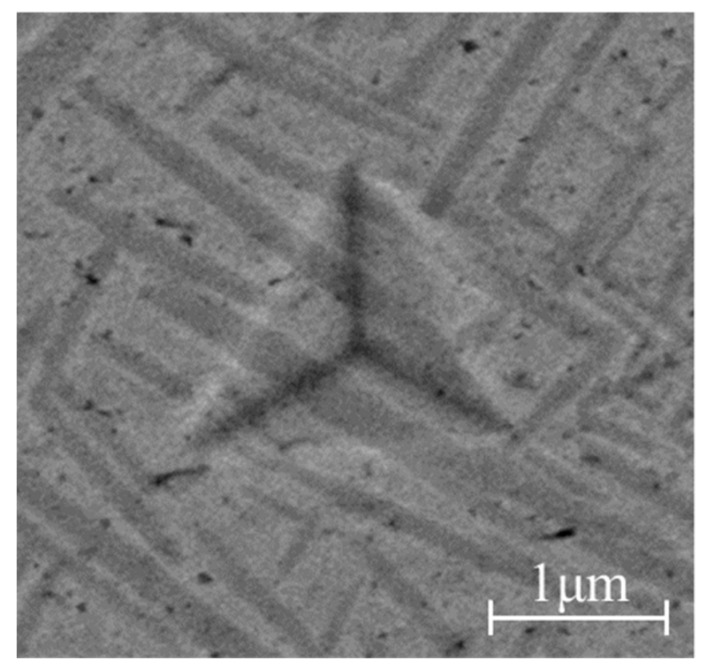
Indentation morphology of TC17 at the indenter load of 200 mN.

Figure 4 shows the indentation morphology of TC17 at room temperature with a loading time of 40 s and a maximum load of 200 mN. It can be observed that the material surface retains noticeable irreversible indentations, which have a strong resemblance to the shape of the indenter. There are evident traces of material being compressed in the vicinity of the indentation.

## 3. Calculation Methods

### 3.1. Indentation Strain Rate

The strain rate field near the indenter tip in the nanoindentation experiment is complex, and the strain rate is not a single value. Therefore, an equivalent strain rate should be introduced in the calculation process. For the Berkovich diamond indenter, according to its geometric similarity, the indentation strain rate can be written following the scaling relations [24]:

(1) $$\dot{\varepsilon }=\frac{\dot{h}}{h}=\frac{1}{h}\frac{dh}{dt}$$

where h is the instantaneous indentation displacement during the holding stage and $\dot{h}$ is the indentation depth rate.

Additionally, h  is calculated by first fitting the h*-*t curve during holding time with an empirical law [25].

(2) h=hi+a(t−ti)12+b(t−ti)14+c(t−ti)18

where t is creep time and $h_{i}$, $t_{i}$, a, b and c are the best-fit parameters.

### 3.2. Indentation Stress

According to the Oliver and Pharr’s model [26], microhardness H can be obtained as

(3) $$H=\frac{P_{max}}{A_{c}}$$

where $P_{max}$ is the holding load and $A_{c}$ is the projected contact area of the Berkovich tip.

**Figure 5:**
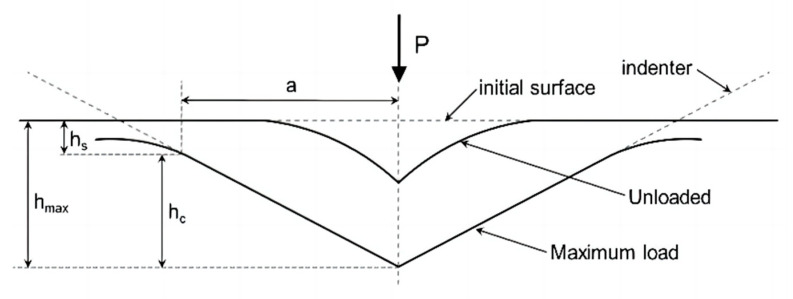
A schematic representation of a section through an indentation showing various quantities used in the analysis.

Figure 5 is the parameter schematic diagram of the indenter loading and unloading, where $h_{s}$ is the displacement of the specimen surface when the indenter is pressed into the specimen. For the standard Berkovich diamond indenter, the relationship between the projected contact area of the Berkovich tip $A_{c}$ and the contact depth $h_{c}$ is as follows [25]:

(4) $$A_{c}=24.56h_{c}^{2}$$

(5) $$h_{c}=h_{max}-0.75\frac{P_{max}}{S}$$

where $h_{max}$ is the total penetration displacement of the indenter at peak load, $P_{max}$ is the peak load at the indenter displacement $h_{max}$, and contact stiffness S is the slope of the initial portion of the unloading curve.

The stress state near the indentation is also complex, and the material is subjected to a complex three-dimensional stress field in the indenting process. Therefore, an equivalent stress value must be introduced in the calculation process, and the calculation of equivalent stress can be expressed as [25,27,28,29]:

(6) $$\sigma =\frac{H}{3}(\frac{h_{max}}{h}{)}^{2}$$

### 3.3. Creep Stress Exponent

Creep stress exponent n is usually used to describe the deformation mechanism in the steady-state creep stage, and it is an index of creep behavior. A power-law creep behavior, which represents the relationship between the creep strain rate and the stress [27], can be described as

(7) $$\dot{\varepsilon }=B\sigma^{n}exp(\frac{-Q}{RT})$$

where B is a temperature-dependent constant, n is the stress exponent, Q is the activation energy, T is the temperature and R is Boltzmann’s constant.

As an indicator for describing the dominant creep mechanism, n is obtained from the slope of the fitted strain rate–stress line [29].

(8) $$n=d(\ln\frac{\dot{h}}{h})∕d(\ln\sigma )$$

## 4. Results and Discussions

### 4.1. Variation of the P-h Curves

**Figure 6:**
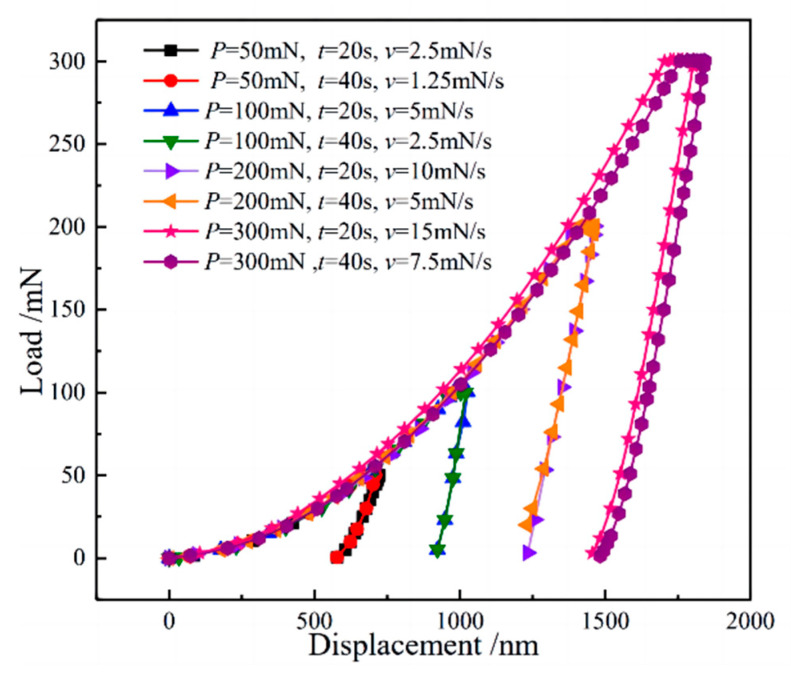
Indentation load–displacement curve during the loading and holding period.

The representative load–displacement (P-h) curves obtained from the nanoindentation experiments are shown in Figure 6. Additionally, P-h curves under various maximum indenter loads are marked by different colors. It can be seen that the P-h curves show similar trends under different loading rates and maximum indenter loads. The surface indentation displacement of the material after unloading is less than the maximum indentation displacement during the holding stage; this reduction in displacement is caused by the elastic recovery of the material, a phenomenon that is typical of indentation in metallic materials. When the maximum load reaches 300 mN, the indentation displacement consistently increases during the holding stage, and notable creep plateaus (i.e., creep displacement during the holding stage) are also evident. The main deformation mechanism during the loading phase is the elastic–plastic deformation of the material. As the maximum load increases, a significant amount of elastic energy is stored near the indenter in the material. This stored energy is then released during the holding phase of the test, causing a substantial increase in the associated plastic deformation and ultimately resulting in an enlarged indentation size. During the unloading process, the elastic recovery rates vary depending on the elastic energy stored prior to unloading for different maximum loads. As a result, the P-h curves exhibit varying unloading slopes.

Table 2 lists the indentation displacement when different load modes are loaded to the maximum load in the test. It can be seen that under the same maximum load, the difference in indentation displacement obtained at different loading rates is small, with a difference of less than 3% of the total indentation displacement. Compared with uniaxial tension or compression experiments, the maximum shear stress of nanoindentation experiments may exceed the yield stress of the sample at a very small displacement, which is the intrinsic reason for the creep phenomenon of many materials through nanoindentation at room temperature. For the four maximum loads taken in this study, the P-h curves basically coincide under different loading rates, and the indentation depth gradually increases with the increase in maximum load. The results show that when the loading rate falls within the range of 1.25 to 15 mN/s, and the maximum load fluctuates between 50 and 300 mN, it is observed that there is no significant impact on the correlation between indentation displacement and load. The actual indentation displacement is solely determined by the maximum load and the duration of the applied force.

### 4.2. Nanoindentation Creep Behavior

**Figure 7:**
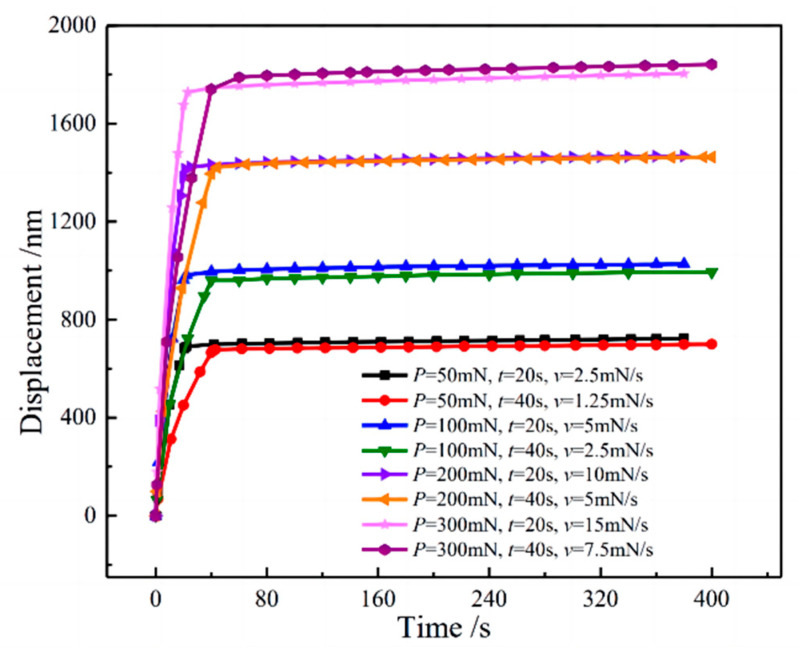
Indentation displacement–time curve during the loading and holding period.

Under different loading modes, the curves of indentation displacement and time in the loading and holding stages of TC17 titanium alloy are shown in Figure 7. It can be seen that the creep displacement curves under different loading rates and maximum loads have the same trend, and combined with the values in Table 2, we can see that there is little difference in indentation displacement when different loading rates are loaded to the same load. The results indicate that loading at different loading rates to the same maximum load has a minimal effect on indentation displacement.

**Figure 8:**
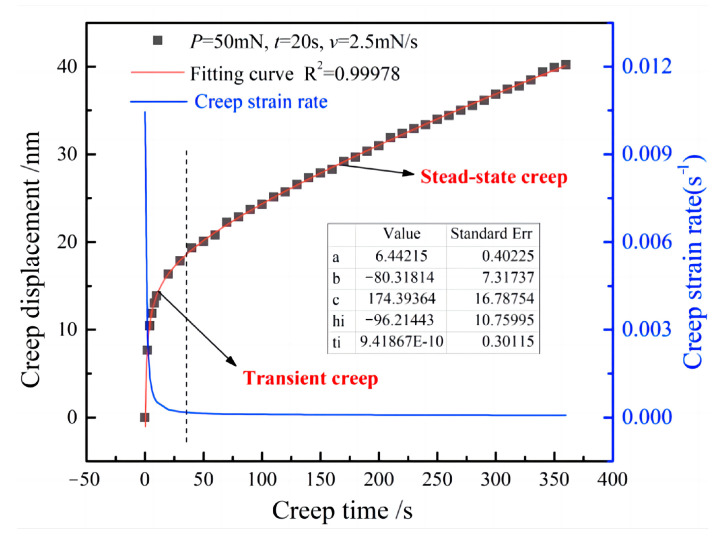
Experimental and fitted creep displacement, creep strain rate and time curve in the loading stage.

When the maximum loading load is 50 mN and the loading time is 20 s, the creep displacement and time curve fitted by (1) are shown in Figure 8. In order to facilitate subsequent comparison, the indentation displacement and time in the initial stage of load retention are zeroed. It can be found from the figure that the experimental data are highly consistent with the fitting curve. The fitting degree correlation index $R^{2}$ of the curve is 0.99978, indicating a remarkably high degree of fitting accuracy. Therefore, the corresponding relation curve between creep strain rate and time can be drawn according to the fitting curve. According to the variation of creep strain rate in the figure, the creep curve can be divided into two distinct stages: transient creep and steady-state creep. Except for the third stage, the creep curve obtained via the nanoindentation test is very similar to the typical creep curve obtained via the tensile test. This can be explained by the fact that the material would not calamitously fail in the nanoindentation test.

**Figure 9:**
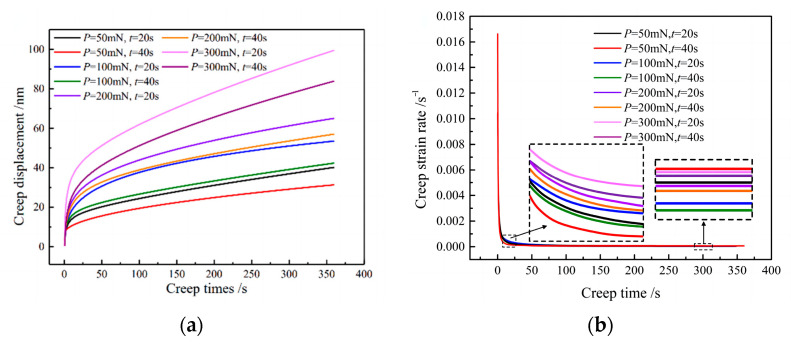
Comparison of fitted curves of (**a**) creep displacement, (**b**) creep strain rate vs. holding time under different loading conditions for TC17.

Figure 9a shows the fitting curves of creep displacement and time under different load modes in the holding stage. It can be seen that all creep curves can be divided into two stages. In the transient creep stage, the creep displacement increases rapidly. Then, in the steady-state creep stage, the creep displacement increases slowly or approximately linearly with time. The primary cause of this phenomenon is the release of elastic deformation accumulated during the loading stage in the initial phase of load holding. At this point, the elastic deformation transitions to plastic deformation, and the creep deformation intensifies. However, as the holding time extends, the creep displacement of the material transitions from an unstable creep stage to a steady creep stage. This transition is due to the increased work hardening and dislocation density. As a result, the creep curve exhibits a stable state in the later stage of load holding. At the same loading time, the greater the maximum load, the greater the indentation displacement in the load-retaining stage. The main reason is that the greater the load, it will not only cause greater elastic–plastic deformation of the material, but also cause greater creep deformation of the material in the load-retaining stage. For the same maximum load, the shorter the loading time, the greater the creep displacement in the holding stage. This is mainly due to the shorter the loading time, wherein the material will have a large amount of elastic energy accumulation in the loading process. Therefore, the material will have greater elastic–plastic deformation in the holding stage. Simultaneously, there will be a higher degree of creep deformation evident during the initial holding stage, which ultimately leads to a larger indentation displacement. As the loading time progresses, the creep deformation of the indentation under different loading methods transitions from the rapid and unstable creep stage to the slower and more stable steady-state creep stage.

Figure 9b shows the variation in creep strain rate over time during the holding stage under different loading modes. Initially, during the early stage of holding, the creep strain rate experiences a rapid decrease, which is attributed to transient creep. As the creep time progresses, the creep strain rate gradually decreases until it reaches a stable value, indicating steady-state creep. This trend change is due to the decrease in strain rate caused by the increase in dislocation density in material hardening and further plastic deformation during creep. Over time, the material not only undergoes hardening but also softening. Ultimately, when equilibrium is achieved, the strain rate approaches a stable value. For the same loading time, the larger the maximum load, the larger the creep strain rate in the holding stage, mainly because the larger load will not only cause greater elastic–plastic deformation of the material but also in the holding stage to make the material undergo greater creep deformation, which leads to a greater creep strain rate. Conversely, for a given maximum load, a shorter loading time in the initial phase leads to an increased creep strain rate during the holding stage. This can be attributed to the fact that the shorter loading time causes a build-up of elastic energy near the indenter, which is not released promptly. As a result, the material undergoes more significant elastic–plastic deformation during the holding period, coupled with a higher degree of creep deformation in the early stage. Therefore, the creep strain rate is greater.

**Figure 10:**
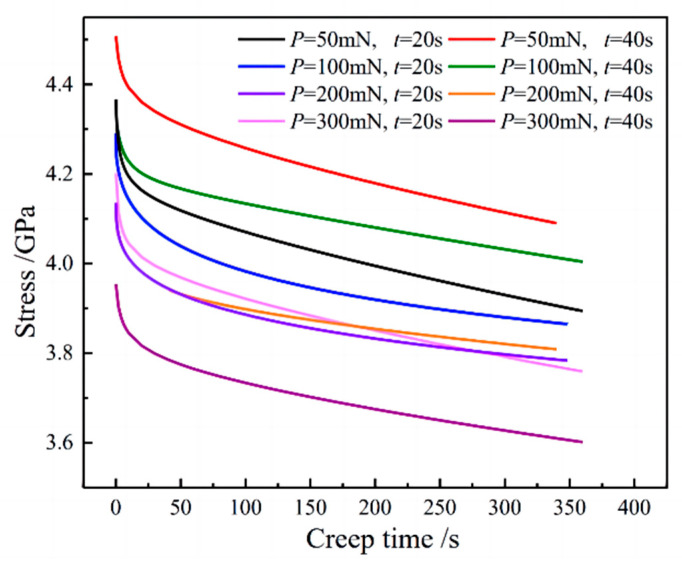
Indentation stress-time curve during the holding period.

According to Equation (6), the variation in material stress with time in the holding stage under various loading modes is calculated, as shown in Figure 10. It can be seen that the material stress decreases with the increase in holding time, which is due to the fact that the indentation displacement of the material continues to increase during the holding stage, making the material stress decrease. Furthermore, with the same loading time, the stress value decreases as the maximum load increases. This phenomenon can be primarily attributed to the larger indentation displacement caused by higher loads, resulting in a decrease in material stress.

### 4.3. Creep Stress Exponents

The creep stress exponent is a significant parameter utilized in the analysis of creep behavior, serving to characterize both creep stability and the primary creep mechanism. The value of the creep stress exponent can provide insight into the deformation mechanisms exhibited by materials during the holding period. Specifically, when the exponent (n) ranges from 1 to 2, the creep deformation under low stress primarily involves diffusion creep and grain boundary sliding mechanisms. Conversely, if dislocation movement predominates the creep deformation, n generally exceeds 3.

In this test, the data are fitted and analyzed according to Equation (8) to determine the stress exponent. The objective of this paper is to measure the stress exponent of the material during the steady-state creep stage. However, it is important to note that the indentation displacement undergoes significant changes during the initial stage of holding time. Such fluctuations in displacement data can lead to substantial errors. To enhance the accuracy of the final fitting results, the fitting process selects relatively stable data from the later stage of holding time to calculate the creep stress exponent.

**Figure 11:**
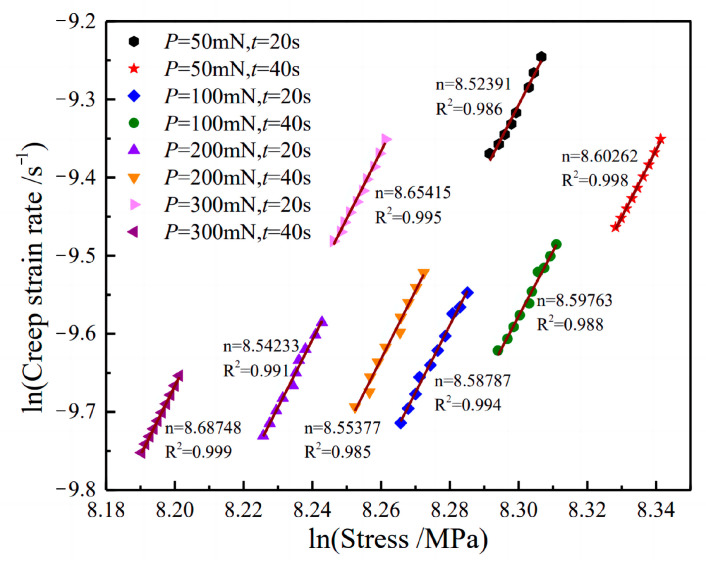
Double logarithmic plot of creep strain rate vs. stress for TC17 alloy.

Figure 11 shows the creep stress exponent of TC17 under different loads and loading times. The creep stress exponent is determined by the slope of the $\ln\dot{\varepsilon }$-ln⁡σ curve. It can be observed that the slopes of the eight curves remain relatively consistent across different loading conditions. The slopes of the fitted curves range from 8.524 to 8.687, with an $R^{2}$ value greater than 0.98 for each curve in the figure. This demonstrates the high accuracy of the curve fitting and confirms the successfulness of the indentation test.

The results show that when the loading rate is within the range of 1.25 to 15 mN/s, and the maximum load fluctuates between 50 and 300 mN, it does not have a significant impact on creep stress exponent of TC17 titanium alloy at room temperature. This is similar to the results in two Pb-Sb alloys, where the stress exponent values are independent of the loading conditions in hardness testing [22]. By conducting nanoindentation tests on TC17 titanium alloy, we can gain valuable insights into its microstructural and mechanical properties, which are crucial for optimizing its performance and developing new applications.

## 5. Conclusions

In this paper, the effect of different maximum loads and loading rates on the creep stress exponent of TC17 alloy at room temperature was studied by the nanoindentation test method. The main conclusions are as follows:

(1) The indentation displacement-load curve obtained by applying different loading rates to the indenter to reach the same maximum load is essentially coincident. Under the same maximum load, the difference in indentation displacement obtained at different loading rates is small, with a difference of less than 3% of the total indentation displacement. This indicates that when the loading rate falls within the range of 1.25 to 15 mN/s, and the maximum load fluctuates between 50 and 300 mN, the indentation displacement is only determined by the maximum load and is negligibly influenced by the loading rate.

(2) By applying different loading rates to the indenter and reaching different maximum loads, the final results show that the obtained stress exponent from different experimental processes are basically consistent, with very small differences. This indicates that when the loading rate is within the range of 1.25 to 15 mN/s, and the maximum load fluctuates between 50 and 300 mN, it does not have a significant impact on the experimental results.

(3) Through the indenter was held under the maximum load for a duration of 360 s, the material in the vicinity of the indenter undergoes corresponding creep deformation, manifested by the indentation depth of the indenter changes with time. By analyzing the relationship between the rate of change of indentation displacement and indentation stress through the late load-holding period, the creep stress exponent distribution range of TC17 titanium alloy at room temperature is determined to be between 8.524 and 8.687.

## Figures and Tables

**Table 1:** The chemical composition of TC17 alloys (in wt.%).

Main Component (wt.%)						Impurities (wt.%)		
Ti	Al	Sn	Zr	Mo	Cr	C	N	O
Bal.	4.9	2.0	1.9	4.2	4.2	0.02	0.02	0.1

**Table 2:** Indentation displacement under different maximum loads.

Maximum Load $\left(F_{max}/\mathrm{mN}\right)$	Loading Time (t/s)	Loading Rate $\left(v/\mathrm{mN}\cdot s^{-1}\right)$	Indentation Displacement (nm)
50	20	2.5	682
50	40	1.25	672
100	20	5	978
100	40	2.5	968
200	20	10	1403
200	40	5	1406
300	20	15	1711
300	40	7.5	1758

## Data Availability

Not applicable.
